# Linking actions and memories: Probing the interplay of action-effect congruency, agency experience, and recognition memory

**DOI:** 10.3758/s13421-024-01644-2

**Published:** 2024-10-09

**Authors:** Marcel R. Schreiner, Shenna Feustel, Wilfried Kunde

**Affiliations:** Julius-Maximilians-Universität Würzburg, Röntgenring 11, 97070 Würzburg, Germany

**Keywords:** Action control, Sense of agency, Memory, Recognition

## Abstract

Adult humans experience agency when their action causes certain events (sense of agency). Moreover, they can later remember what these events were (memory). Here, we investigate how the relationship between actions and events shapes agency experience and memory for the corresponding events. Participants performed actions that produced stimuli that were either congruent or incongruent to the action while memory of these stimuli was probed in a recognition test. Additionally, predictability of the effect was manipulated in Experiment 1 by using either randomly interleaved or blocked ordering of action-congruent and action-incongruent events. In Experiment 2, the size of the action space was manipulated by allowing participants to choose between three or six possible responses. The results indicated a heightened sense of agency following congruent compared to incongruent trials, with this effect being increased given a larger available action space, as well as a greater sense of agency given higher predictability of the effect. Recognition memory was better for stimuli presented in congruent compared to incongruent trials, with no discernible effects of effect predictability or the size of the action space. The results point towards a joint influence of predictive and postdictive processes on agency experience and suggest a link between control and memory. The partial dissociation of influences on agency experience and memory cast doubt on a mediating role of agency experience on the relationship between action-effect congruency and memory. Theoretical accounts for this relationship are discussed.

Through actions, agents cause subsequent effects in their environment, of which many are predictable from previous experience. For example, if one pushes a glass across a table, one would expect that the distance of the glass to oneself increases. However, much of an agent’s environment lies in an uncontrollable state, such that an agent’s actions have no bearing on the environmental outcomes. A sense of agency (SoA) allows us to distinguish between environmental changes that are caused by the self from those that are not. The SoA is the experience of controlling environmental outcomes through one’s actions (Haggard, 2017; Haggard & Tsakiris, 2009). It has been suggested that a SoA emerges when an action outcome predicted by a motor command (Blakemore & Frith, 2003; Frith, 2005; Haggard, 2017) or an agent’s perceptual goal (Niziolek et al., 2013; Verschoor & Hommel, 2017) matches the environmental outcome. This predictive component of the SoA occurs before the action (Haggard, 2017) and is facilitated by increased predictability of effects (Liesner et al., 2020; Ma et al., 2019) and action selection fluency (Chambon et al., 2014; Wenke et al., 2010).

Besides this predictive component of the SoA (Beck et al., 2017; Schwarz et al., 2018, 2022), agents can also experience SoA for outcomes they did not predict (e.g., Ramachandran & Rogers-Ramachandran, 1997), and this may foster the acquisition of novel action-effect contingencies. Thus, there is likely also a postdictive component to the SoA, such that agents employ explicit reasoning processes to determine whether their action caused an effect in the environment (Liesner et al., 2020; Synofzik et al., 2008; Wegner, 2003; Wegner & Wheatley, 1999). Postdictive SoA experience is shaped by various factors. One factor doing so is the congruency between action and effect. For example, in a study by Ebert and Wegner (2010), participants moved a joystick back and forth, which triggered either a back or a forth movement of a picture on a screen. The ensuing movement of the picture varied from trial to trial, and was thus unpredictable prior to moving the joystick. Still, participants showed higher levels of SoA when the movement of the picture was spatially congruent to the movement of the joystick (e.g., forward movement of the joystick producing a forward movement of the picture) than when it was not (e.g., forward movement of the joystick producing a backwards movement of the picture). Thus, although picture movements were objectively unpredictable in the experimental context, SoA was nevertheless higher with spatial congruency of action and picture movement. Therefore, this influence of action-effect congruency can be said to be postdictive, in the sense that it could not rest on predictions possible in the particular experimental context, while perhaps still expressing pre-experimentally established knowledge of how hands and objects typically move (e.g., pushing objects typically moves them away). In addition, SoA tends to be higher if agents can freely choose an action than when they are instructed on which to choose (Barlas & Obhi, 2013; Borhani et al., 2017; Sebanz & Lackner, 2007; Sidarus et al., 2017; Villa et al., 2021), although this may not always be the case and may depend on the task and measurement of SoA (Schwarz et al., 2019). Furthermore, SoA increases with an increasing number of possible actions which the agent can choose from Barlas et al. (2017); Barlas and Kopp (2018); Barlas and Obhi (2013).

Actions and control are also related to memory. For example, the physical enactment of an event leads to better memory than merely reading instructions (i.e., the enactment effect, Engelkamp, 1986; Roberts et al., 2022). Being an actor rather than an observer in an episode also causes participants to exhibit stronger hippocampal reactions to expectation violations (Jainta et al., 2022). Even simple key-press actions can lead to enhanced recognition memory in a Go/No-Go task (Chiu & Egner, 2015; Yebra et al., 2019). Being able to exert control during an event also improves memory. In a study by Ruiz et al. (2023), characters were contestants in a game show and had to select one out of three doors to win a prize. Participants could either choose the door for the contestant themselves (i.e., were able to exert control) or they were instructed to choose a specific door. Memory for contestants, associations between contestants and doors, and associations between contestants and prizes was better if participants could exert control over the event. In addition, being able to exert control over learning content can improve memory, as demonstrated by better memory for items that participants generated themselves compared to items they only read (i.e., the generation effect, Jacoby, 1978; Slamecka & Graf, 1978).

It has further been suggested that memory is better for stimuli associated with a stronger SoA as a result of spatial congruency and temporal contiguity between an action and its effect (Hon & Yeo, 2021). In this study, participants made self-decided and -initiated up or down arrow key presses to move a box. After the box stopped moving, a word appeared inside of it and participants subsequently rated their SoA over the box movement. The spatial congruency and temporal contiguity of the action and the box movement were manipulated by making the box move either in the direction of the key press or in the opposite direction and by initiating the movement after a delay of either 100 or 900 ms after the action. While both spatial congruency and temporal contiguity yielded a higher SoA, they also yielded better memory (hit rates) for the presented words in a later recognition test. However, Tsuji and Imaizumi (2022) conducted a similar study, but could only replicate the effect of spatial congruency on the SoA, but not the effect of spatial congruency or SoA on memory. Thus, the robustness of this effect remains unclear.

In the current research, we aim to investigate how congruency between an action and ensuing perceptual changes in the environment shape the SoA and memory for the produced event. We thereby aim to replicate the findings by Hon and Yeo (2021) and extend them in several aspects by distinguishing between predictive and postdictive components of the SoA and investigating influences of the size of the available action space (i.e., the number of possible actions).

## Experiment 1

In Experiment 1, we aimed to replicate the effect of action-effect congruency on SoA and recognition memory as found by Hon and Yeo (2021), while extending the investigation to differentiate between different contributions of predictive and postdictive components of the SoA, and to consider effects of changes in control. Closely following the procedure by Hon and Yeo (2021), participants’ up or down key presses moved a box in an either spatially congruent or incongruent direction (congruency condition). After the box stopped moving, a word appeared inside the box and these words were later tested in an old/new recognition test. We only used a short delay of 100 ms for the onset of the box movement, thus not considering temporal action-effect contiguity. In addition, we varied the predictability of the box movement, by either ordering congruent and incongruent trials randomly or blocking them, such that there was one block with congruent and one block with incongruent trials (order condition). Thus, the blocked order condition created a high predictive environment, in which the box’s movement was highly predictable, whereas the random order condition created a low predictive environment, in which the box’s movement was unpredictable.

First, we expected the SoA to be higher for stimuli associated with an effect congruent to the action than for stimuli associated with an effect incongruent to the action (Hypothesis 1), as has been repeatedly found in previous studies (Hon & Yeo, 2021; Liesner et al., 2020; Tsuji & Imaizumi, 2022). Second, we expected this to also be the case for recognition memory (Hypothesis 2), as found by Hon and Yeo (2021). Third, if the effect of action-effect congruency on recognition memory is primarily mediated by the predictive component of the SoA, it should be stronger when the effect is predictable from the task structure (i.e., in the blocked order condition). However, if the effect is primarily mediated by the postdictive component of the SoA, it should emerge no matter whether the effect is predictable in the current context or not (i.e., in both the random and blocked order conditions, Hypothesis 3). Fourth, we investigated whether the influence of action-effect congruency on recognition memory is sensitive to changes in control (Hypothesis 4), which we report in Appendix A. The experiment’s design, hypotheses, and analysis plan were preregistered at https://doi.org/10.17605/OSF.IO/SF6JE.

### Methods

#### Participants

Participants were recruited from the participant pool of the University of Würzburg and received a compensation of 5€. They were prescreened to be native German speakers and to have normal or corrected-to-normal vision. An a priori power analysis using G*Power 3.1 (Faul et al., 2009) for detecting effects of congruency condition within the two order conditions (Hypotheses 1–3) given a moderate effect size (*f* = .25) with 90% power yielded a desired sample size of 46 participants. However, an a priori power simulation using the R package *simr* (version 1.0.7, Green & MacLeod, 2016) for testing the effect of change in control (Hypothesis 4, two-tailed testing) with 90% power based on data from a pilot study (*N* = 20) yielded a desired sample size of 60 participants in the random order condition. Because we aimed for an equal number of participants in the two order conditions, we collected 60 participants per order condition, for a total of 120 participants. Two participants had to be excluded. One because they suggested their data should not be used and another due to exhibiting a biased selection of response keys in the learning phase (proportion of up key presses greater than .8 or lower than .2, cf. Naefgen et al., 2018; Tsuji & Imaizumi, 2022). This was done to screen out participants with low effort responding and to ensure variability in responses. Thus, the final sample consisted of 118 participants (57 participants in the random order condition and 61 participants in the blocked order condition).^1^ The mean age of participants was 28.0 years (*SD* = 10.3) in the random order condition (38 female, 51 right-handed) and 25.4 years (*SD* = 8.1) in the blocked order condition (51 female, 56 right-handed, 1 ambidextrous).

#### Design

The experiment employed a 2 (congruency condition: congruent vs. incongruent) $\times$ 2 (order condition: random vs. blocked) mixed design, with congruency condition being manipulated within-subjects and order condition being manipulated between-subjects. In the congruent condition, the box moved in the direction indicated by the participants’ key presses (e.g., up after an up arrow key press) and in the incongruent condition, it moved in the opposite direction. In the random order condition, congruency was manipulated on a trial-by-trial basis (i.e., trial order was randomized) and in the blocked order condition, it was manipulated in a blocked manner, such that all congruent trials occurred before all incongruent trials or vice versa. The order of the two blocks (congruent or incongruent trials first) was counterbalanced across participants. For an exploratory analysis of the effects of block order see Appendix B.

#### Apparatus and stimuli

Participants sat in front of a 24-inch monitor with a resolution of 1920 px $\times$ 1080 px and a refresh rate of 100 Hz, with a viewing distance of approximately 60 cm. They responded using a standard QWERTZ keyboard. The experiment was implemented using OpenSesame 3.3.5 (Mathôt et al., 2012). Stimuli were 120 German nouns taken from the Berlin Affective Word List Reloaded (Võ et al., 2009). Words were selected based on the following criteria: Neutral emotional valence (mean ratings between -0.5 and 0.5 on a seven-point rating scale ranging from -3 to 3), mean imaginability ratings between 2.5 and 5.5 (measured on a seven-point rating scale ranging from 1 to 7), mean arousal ratings smaller than 4 (measured on a five-point rating scale ranging from 1 to 5), and same length of all words (five letters). Of the remaining words, the three least and ten most frequently used ones were excluded.

**Fig. 1:**
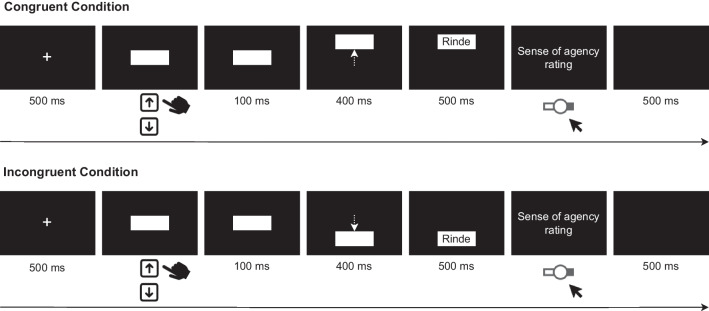
Schematic depiction of the learning phase in Experiment 1

#### Procedure

The experimental procedure closely followed the one by Hon and Yeo (2021) (Experiment 2). Participants were randomly assigned to the order conditions, such that they would be equally distributed across conditions. The experiment consisted of a learning phase and a test phase. In the learning phase, participants were first presented with a fixation cross for 500 ms and then a white box (width = $6^{\circ }$, height = $3^{\circ }$ when viewed from a distance of 60 cm) on a black background. They then made self-decided and -initiated up or down arrow key presses, but were instructed to avoid any bias in the selection of response keys (specifically, to try to use both keys approximately equally often across trials). After a delay of 100 ms (the shorter delay condition in Hon and Yeo (2021)), the box moved $5.14^{\circ }$ upward or downward for 400 ms, either in the direction of the key press (congruent condition) or in the opposite direction (incongruent condition). After the box stopped moving, a word (height = $0.75^{\circ }$) appeared superimposed on the box for 500 ms. Participants’ SoA over the box movement was then assessed by asking them to rate how much they felt their key press to have controlled the box’s movement. Responses were made using a continuous slider ranging from 0 (*no control*) over 50 (*unsure*) to 100 (*full control*). Each trial was followed by a 500-ms intertrial interval during which a blank screen was shown. A schematic depiction of the learning phase is given in Fig. 1. The learning phase consisted of 80 trials (40 per congruency condition). Eighty words were randomly drawn from the pool of 120 words and were randomly assigned to the congruency conditions. The remaining 40 words served as novel foils in the test phase. Before the learning phase, participants conducted four learning trials (two per congruency condition) in which the word “Wort” (“word” in German) was shown to familiarize them with the task.

Directly after the learning phase, the test phase started, during which participants conducted an incidental old/new recognition memory test. Forty words (20 per congruency condition) were randomly drawn from the ones used in the learning phase. These were mixed with 40 novel foils. Thus, the test phase consisted of 80 trials. Each trial consisted of a 500-ms fixation cross followed by a word presented in the screen center. Participants indicated whether they thought the word was new (i.e., did not occur in the learning phase) by pressing the Y key on the keyboard, or old (i.e., occurred in the learning phase) by pressing the M key. In the left and right of the lower half of the screen, the words “neu” (“new” in German) and “alt” (“old” in German) were presented to facilitate participants’ understanding of the correct key-response mapping. Each trial was preceded by an intertrial interval of 500 ms. The trial order was randomized. Before the test phase, participants conducted three practice trials in which the word “Wort” (“word” in German) was shown to familiarize them with the task. After the test phase, participants provided some demographic information and could specify reasons that might warrant the exclusion of their data from the analyses and give comments regarding the study. Finally, they were thanked and debriefed.

### Results

All analyses were conducted in R 4.3.1 (R Core Team, 2023) and we used the R packages *papaja* (version 0.1.2, Aust & Barth, 2023) and *tinylabels* (version 0.2.4, Barth, 2023) for reporting. We used the conventional significance level of $\alpha$ = .05 for all analyses. SoA ratings were divided by 100 to recode them to the interval [0, 1]. Analyses of Variance (ANOVAs) were performed using the package *afex* (version 1.3-0, Singmann et al., 2023). Planned contrasts and post-hoc pairwise comparisons were tested using the package *emmeans* (version 1.8.9, Lenth, 2023). Effect sizes for planned contrasts and post-hoc pairwise comparisons, as well as confidence intervals for ANOVA effect sizes, were estimated using the package *effectsize* (version 0.8.6, Ben-Shachar et al., 2020). (Generalized) mixed linear models (see Goldstein, 2011; Hoffman & Rovine, 2007) were fit using the packages *lme4* (version 1.1-35.1, Bates et al., 2015) and *lmerTest* (version 3.1-3, Kuznetsova et al., 2017).

#### Sense of agency

**Fig. 2:**
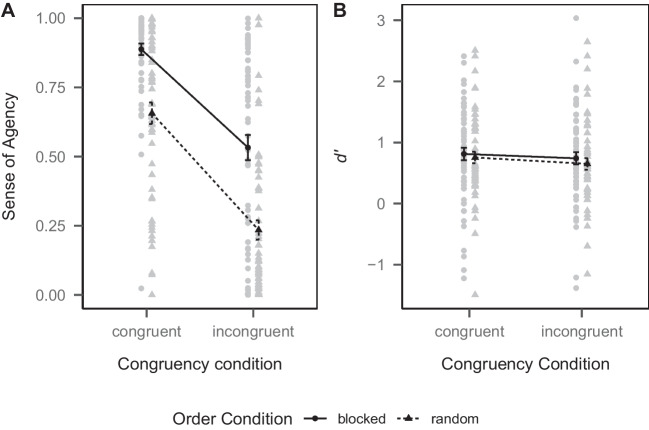
Mean sense of agency ratings (**A**) and mean *d’* values (**B**) by congruency and order condition in Experiment 1. *Notes*. *Black shapes* depict group-level means, *gray shapes* depict person-level means. *Error bars* depict ± *SEM*

Mean SoA ratings are depicted in Fig. 2A. To test whether SoA was higher in the congruent than in the incongruent condition (Hypothesis 1), we first performed a 2 (congruency condition) $$\times $$ 2 (order condition) mixed-design ANOVA on the SoA ratings and then tested planned contrasts (one-tailed testing). There was a main effect of congruency condition ($$F(1, 116) = 165.33$$, $$ MSE = 0.05$$, $$p < .001$$, $$\hat{\eta }^2_p = .588$$, 95% CI [.48, .67]). Planned contrasts revealed that SoA was higher in the congruent condition (*M* = 0.78, *SD* = 0.26) than in the incongruent condition (*M* = 0.39, *SD* = 0.34, $$\Delta M = 0.78$$, 95% CI $$[0.68, \infty ]$$, $$t(116) = 12.86$$, $$p < .001$$, $$\hat{d}$$ = 1.19, 95% CI [0.95, 1.43]), supporting Hypothesis 1. This was the case both within the random order condition ($$\Delta M = 0.42$$, 95% CI $$[0.35, \infty ]$$, $$t(116) = 9.71$$, $$p < .001$$, $$\hat{d}$$ = 0.90, 95% CI [0.68, 1.12]) and within the blocked order condition ($$\Delta M = 0.36$$, 95% CI $$[0.29, \infty ]$$, $$t(116) = 8.46$$, $$p < .001$$, $$\hat{d}$$ = 0.79, 95% CI [0.58, 0.99]). These results were mirrored when analyzing SoA on the trial instead of on the aggregate level using a mixed linear model with random person intercepts. Furthermore, there was a main effect of order condition ($$F(1, 116) = 41.83$$, $$ MSE = 0.10$$, $$p < .001$$, $$\hat{\eta }^2_p = .265$$, 95% CI [.14, .39]). SoA was higher in the blocked order condition (*M* = 0.71, *SD* = 0.33) than in the random order condition (*M* = 0.45, *SD* = 0.35). There was no interaction of congruency condition and order condition ($$F(1, 116) = 1.22$$, $$ MSE = 0.05$$, $$p = .272$$, $$\hat{\eta }^2_p = .010$$, 95% CI [.00, .07]).

#### Recognition memory

To test whether recognition memory was better for words in the congruent compared to the incongruent condition (Hypothesis 2) and whether this effect was present in both the random and blocked order condition (Hypothesis 3), we computed *d’* from signal detection theory from the hit and false alarm rates in the recognition test, separately for the congruent and incongruent condition, as a measure of recognition performance. *d’* is a sensitivity index that controls for participants’ response bias. Because there were participants with extreme proportions of hit or false alarm rates (0 or 1), for which *d’* can not be calculated, we used the loglinear approach (Hautus, 1995) to adjust the rates.^2^ We thus added 1/3 to the number of hits and 2/3 to the number of false alarms (reflecting the ratio of signal-to-noise trials when considering congruent and incongruent trials separately), and 2/3 to the number of signal and 4/3 to the number of noise trials before calculating the hit and false alarm rates. Mean *d’* values are depicted in Fig. 2B. We first performed a 2 (congruency condition) $$\times $$ 2 (order condition) mixed-design ANOVA on *d’* and then tested planned contrasts (one-tailed testing). There was a main effect of congruency condition ($$F(1, 116) = 3.93$$, $$ MSE = 0.12$$, $$p = .050$$, $$\hat{\eta }^2_p = .033$$, 95% CI [.00, .12]). Planned contrasts revealed that recognition memory was better in the congruent condition (*M* = 0.78, *SD* = 0.75) than in the incongruent condition (*M* = 0.70, *SD* = 0.74, $$\Delta M = 0.18$$, 95% CI $$[0.03, \infty ]$$, $$t(116) = 1.98$$, $$p = .025$$, $$\hat{d}$$ = 0.18, 95% CI [0.00, 0.37]), supporting Hypothesis 2. However, this effect was only significant when testing across the two order conditions, but not within either the random order condition ($$\Delta M = 0.10$$, 95% CI $$[0.00, \infty ]$$, $$t(116) = 1.63$$, $$p = .053$$, $$\hat{d}$$ = 0.15, 95% CI [-0.03, 0.33]) or blocked order condition ($$\Delta M = 0.07$$, 95% CI $$[-0.03, \infty ]$$, $$t(116) = 1.17$$, $$p = .123$$, $$\hat{d}$$ = 0.11, 95% CI [-0.07, 0.29]). This does not allow for a clear inference regarding Hypothesis 3. There was neither a main effect of order condition (random order condition: *M* = 0.70, *SD* = 0.71, blocked order condition: *M* = 0.78, *SD* = 0.78, $$F(1, 116) = 0.33$$, $$ MSE = 1.00$$, $$p = .567$$, $$\hat{\eta }^2_p = .003$$, 95% CI [.00, .05]), nor was there an interaction of congruency condition and order condition ($$F(1, 116) = 0.13$$, $$ MSE = 0.12$$, $$p = .721$$, $$\hat{\eta }^2_p = .001$$, 95% CI [.00, .04]).^3^

As a supplemental analysis, we also performed the same tests using (unadjusted) hit rates instead of *d’* as the dependent variable to mirror the analysis by Hon and Yeo (2021). There was a main effect of congruency condition ($$F(1, 116) = 4.88$$, $$ MSE = 0.01$$, $$p = .029$$, $$\hat{\eta }^2_p = .040$$). Planned contrasts revealed that recognition memory was better in the congruent condition (*M* = 0.62, *SD* = 0.20) than in the incongruent condition (*M* = 0.59, *SD* = 0.19, $$\Delta M = 0.06$$, 95% CI $$[0.02, \infty ]$$, $$t(116) = 2.21$$, $$p = .015$$, $$\hat{d}$$ = 0.21, 95% CI [0.02, 0.39]). Here, the effect was also significant within the random order condition ($$\Delta M = 0.04$$, 95% CI $$[0.00, \infty ]$$, $$t(116) = 1.75$$, $$p = .042$$, $$\hat{d}$$ = 0.16, 95% CI [-0.02, 0.35]), but not in the blocked order condition ($$\Delta M = 0.03$$, 95% CI $$[-0.01, \infty ]$$, $$t(116) = 1.37$$, $$p = .087$$, $$\hat{d}$$ = 0.13, 95% CI [-0.06, 0.31]). There was neither a main effect of order condition (random order condition: *M* = 0.59, *SD* = 0.19, blocked order condition: *M* = 0.62, *SD* = 0.20, $$F(1, 116) = 0.57$$, $$ MSE = 0.06$$, $$p = .453$$, $$\hat{\eta }^2_p = .005$$), nor was there an interaction of congruency condition and order condition ($$F(1, 116) = 0.09$$, $$ MSE = 0.01$$, $$p = .760$$, $$\hat{\eta }^2_p = .001$$). These results were mirrored when analyzing hits (1 = hit, 0 = miss) on the trial instead of on the aggregate level using a generalized mixed linear model with a logit link function and random person intercepts. The results therefore largely mirror the ones with *d’* as the dependent variable, but the effects were slightly larger and the difference between the congruent and incongruent condition within the random order condition reached significance when using hit rates as the dependent variable.

### Discussion

In Experiment 1, we found a positive effect of action-effect congruency on the SoA and recognition memory, supporting Hypotheses 1 and 2. The effect of congruency between an action and its effect is in line with earlier findings (Farrer et al., 2008; Hon & Yeo, 2021; Liesner et al., 2020; Tsuji & Imaizumi, 2022; Wegner & Wheatley, 1999). Congruency between an action and subsequently unfolding environmental effects thus appears to be a strong driver of the SoA. In addition, we found a higher SoA when the action effect was predictable (in the blocked order condition) than when it was unpredictable (in the random order condition). This is consistent with Liesner et al. 2020 and, together with the effect of congruency, suggests the independent influence of pre- and postdictive components on the SoA. Finding the effect of congruency to persist in the blocked order condition also suggests that participants did not modify their behavior by selecting the opposite response option in the incongruent block to achieve desired outcomes, as SoA should have also been high in the incongruent block in this case.

By finding a positive effect of action-effect congruency on recognition memory, we could replicate the finding of Hon and Yeo (2021), but not the one of Tsuji and Imaizumi (2022), who did not find such an effect. However, while using the same experimental paradigm, their study included an additional baseline condition, in which the box moved up- or downwards randomly after a jittered delay without a key presses. It is possible that this baseline condition interfered with the perceived contingency between the participants’ action and the subsequent effect. In addition, our study had a much larger sample than the one of Tsuji and Imaizumi (2022), and was thus much higher powered. In fact, we found the effect of action-effect congruency on recognition memory to be quite small, and much smaller than the effects reported by Hon and Yeo (2021). Hon and Yeo (2021) used hit rates as the dependent variable, which do not take possible response biases of the participants into account. For example, participants who adopt a liberal response criterion and respond “old” to most items will naturally have high hit rates, but also high false alarm rates. This is not apparent when only considering hit rates. We thus used the sensitivity index *d’* from signal detection theory to take participants’ response biases into account. In our own supplemental analysis as well, effects tended to be larger when using hit rates instead of *d’* as the dependent variable, suggesting that disregarding response biases may inflate the effect of action-effect congruency on memory.

In addition, we only reliably found the effect when collapsing over the blocked and random order conditions (except for the supplemental analysis of hit rates, in which the effect was also significant within the random order condition). This may be due to the rather small effect requiring a large sample size to be detected, but it prevents us from drawing strong conclusions regarding Hypothesis 3. However, if effects on recognition memory were mediated by SoA, one would expect that recognition memory would be better in the blocked order condition, in which SoA was higher. This was not the case. The higher SoA in the blocked order condition did not translate into significantly better recognition memory in that condition. It is therefore possible that the effect of action-effect congruency on recognition memory is not mediated by SoA, but that action-effect congruency may independently affect both SoA and recognition memory.

In Experiment 2, we set out to test the robustness of the effect of action-effect congruency on SoA and recognition memory. We further investigated effects of the size of the available action space and distinguished between different recognition memory processes.

## Experiment 2

In Experiment 2, we used a different experimental paradigm and switched to a less controlled online setting to test the robustness of the previously found effect of action-effect congruency on SoA and recognition memory. In addition, we investigated the effect of the size of the available action space (i.e., the number of response options to choose from). Participants were presented line drawings (icons) which they could colorize by selecting a color. They could either choose between three colors (small set condition) or six colors (large set condition), which thus constituted a smaller or larger available action space. After selecting a color, the icon was either colored in the selected color (congruent condition) or in a color randomly chosen from the non-selected response options (incongruent condition). Icons were later tested in a recognition test. Recognition memory performance reflects two distinct processes: recollection (i.e., conscious and detailed memory of previously encountered events, including stimuli and the context in which they occurred) and familiarity (i.e., the feeling that an event or stimulus was previously encountered, Yonelinas, 2002). In Experiment 2, we aimed to distinguish between these processes using the remember/know procedure (Gardiner, 1988; Tulving, 1985), in which remember judgments are thought to reflect recollection, and know judgments are thought to reflect familiarity. Thus, in the recognition test, participants could indicate whether they remembered the icon, merely knew that it occurred in the learning phase, or whether it was new (see also Tsuji & Imaizumi, 2022).

First, we aimed to replicate the effect of action-effect congruency on SoA and therefore expected the SoA to again be higher for stimuli associated with an effect congruent to the action than for stimuli associated with an effect incongruent to the action (Hypothesis 1a). A facilitating effect of the size of the available action space has been found in previous studies (Barlas et al., 2017; Barlas & Kopp, 2018; Barlas & Obhi, 2013). For example, Barlas and Obhi (2013) found the SoA to increase monotonically with the number of response options increasing from one (forced-choice) to four. Considering these previous findings, we expected the effect of action-effect congruency to be modulated by the size of the available action space. We thus expected the difference in SoA between congruency and incongruency of an action and its effect to be larger given a larger available action space (Hypothesis 1b). Second, we expected this pattern to also be reflected in recognition memory. This is because, if one assumes that the effect of action-effect congruency on memory is driven by the SoA, a modulation of the SoA by the size of the available action space should also influence memory. In addition, memory may benefit from items being more uniquely coupled with an action, as actions may then serve as more effective retrieval cues (Raaijmakers & Shiffrin, 1980, 1981). In typical paradigms investigating the enactment effect (e.g., Engelkamp, 1986) for example, actions are uniquely paired with other information and we aimed to approximate this with a larger available action space. However, such a beneficial influence may depend on the strength of the encoded association between action and effect, which may be stronger given action-effect congruency. Thus, we expected that recognition memory is better for stimuli associated with action-effect congruency than stimuli associated with action-effect incongruency (Hypothesis 2a) and that the difference in recognition memory for stimuli associated with action-effect congruency or incongruency is larger given a larger available action space (Hypothesis 2b). Finally, given a larger available action space, participants may more strongly consider the effects they produce as self-generated and thus more self-relevant. Self-reference effects have mainly been found in recollection (Conway & Dewhurst, 1995; van den Bos et al., 2010). In addition, recollection involves the retrieval of contextual information and therefore, the action or certain effect features may serve as retrieval cues, facilitating memory. We thus expected the effect of the available action space to more strongly influence recollection rather than familiarity (Hypothesis 3). This may also be the case for the effect of action-effect congruency (although this was not preregistered). For an exploratory analysis of the effect of change in control see Appendix A. The experiment’s design, hypotheses, and analysis plan were preregistered at https://doi.org/10.17605/OSF.IO/MBXK4.

### Methods

#### Participants

Participants were recruited from the participant pool of the University of Würzburg and could receive course credit for their participation. They were prescreened to have normal or corrected-to-normal vision and no color vision deficiencies. An a priori power analysis using G*Power 3.1 (Faul et al., 2009) for detecting a main effect for a two-level within-subjects factor (Hypotheses 1a and 2a) or a 2 $\times$ 2 within-between interaction (Hypotheses 1b, 2b, and 3) given a small effect size (*f* = .15) with 90% power yielded a desired sample size of 120 participants (60 per between-subjects condition). To account for possible data exclusions, we oversampled by 15%, thus aiming for a sample size of 138 participants. Ultimately, 145 participants completed the study before data collection was stopped. All participants provided informed consent for participation and publication of their data. Thirty-four participants had to be excluded. One indicated being younger than 18 years. Five reported having experienced technical problems. Further, we excluded 26 participants who chose a single color with a proportion less than half or more than 1.5 times from chance in the learning phase (i.e., $< 1/6$ or $> 1/2$ for the small set condition and $< 1/12$ or $> 1/4$ for the large set condition).^4^ Two other participants were excluded, one due to taking more than 5 s to respond in at least 5% of trials (to avoid heterogeneity in encoding times), and another one due to indicating not having conducted the study properly. Thus, the final sample consisted of 111 participants (62 participants in the small set condition and 49 participants in the large set condition). The mean age of participants was 21.5 years (*SD* = 4.7) in the small set condition (53 female) and 22.3 years (*SD* = 7.0) in the large set condition (44 female).

#### Design

The experiment employed a 2 (congruency condition: congruent vs. incongruent) $\times$ 2 (set size condition: small vs. large) mixed design, with congruency being manipulated within-subjects and set size being manipulated between subjects. In the congruent condition, the coloring of the icon matched the color selected by the participant and in the incongruent condition, it was instead colored with one of the non-selected colors (randomly chosen). In the small set condition, participants could choose between three available colors and in the large set condition they could choose between six available colors.

#### Apparatus and stimuli

The experiment was implemented using lab.js (Henninger et al., 2022) and conducted online. Data collection was managed by JATOS (Lange et al., 2015). Stimuli were two-dimensional icons of objects in the format of scalable vector graphics (SVGs) taken from the Dazzle Line Icons Collection on SVG Repo (Dazzle UI, https://www.svgrepo.com/collection/dazzle-line-icons/). Icons were selected to have similar line thickness and to be sufficiently distinguishable from one another. All icons were of the same size. Two additional icons were used as practice stimuli. In the small set condition, participants could choose between the colors red (hexadecimal color code: #ff0000), green (#00ff00), and blue (#004bff). In the large set condition, they could choose between red, orange (#ff7f00), yellow (#ffdc00), green, blue, and purple (#aa00ff).

**Fig. 3:**
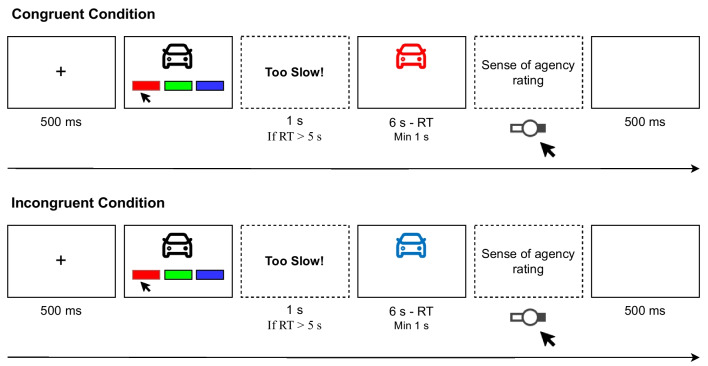
Schematic depiction of the learning phase in Experiment 2 for the small set condition. *Notes*. Screens with *dashed lines* were not always shown. Sense of agency ratings were collected every fourth occurrence of a congruent or incongruent trial, respectively. RT = reaction time, Min = minimum

#### Procedure

Participants were randomly assigned to the set size conditions, such that they would be equally distributed across conditions. This experiment too consisted of a learning phase and a test phase. In the learning phase, participants were first presented with a fixation cross for 500 ms and then a black icon depicting an object on a white background. Below the item, there were three (small set condition) or six (large set condition) buttons with colors in one (small set condition) or two (large set condition) rows. Within a set size condition, colors were always in the same order for all participants. Participants then made a self-decided and -initiated selection of a color by clicking on the corresponding button, but were instructed to avoid any bias in the selection of colors. They were further instructed to make their selection within 5 s. If they were too slow, they received corresponding feedback for 1 s after having made their selection, and the trial was flagged as a timeout. After participants selected a color, the color buttons disappeared and the icon was colored with the selected color (congruent condition) or with a randomly chosen color from the ones not selected by the participant (incongruent condition). The colored icon was displayed for 6 s minus the time it took participants to select a color, but for a minimum of 1s. This was done to align the total presentation duration across trials. Every fourth occurrence of a congruent or incongruent trial, respectively, participants’ SoA over the coloring of the icon was assessed by asking them to rate how much they felt to have controlled the coloring of the object using a continuous slider ranging from 0 (*no control*) to 100 (*full control*). This resulted in ten ratings per congruency condition. Each trial was followed by a 500-ms intertrial interval, during which a blank screen was shown. A schematic depiction of the learning phase is given in Fig. 3. The learning phase consisted of 80 trials (40 per congruency condition). Eighty icons were randomly drawn from the pool of 120 icons and were randomly assigned to the congruency conditions. The remaining 40 icons served as novel foils in the test phase. Before the learning phase, participants conducted two learning trials (one per congruency condition) to familiarize them with the task.

Directly after the learning phase, the test phase started, during which participants conducted an incidental recognition memory test. Forty icons (20 per congruency condition) were randomly drawn from the ones used in the learning phase. These were mixed with 40 novel foils. Thus, the test phase consisted of 80 trials. In each trial, a black icon was presented in the screen center with three buttons underneath it. We used the remember/know procedure (Gardiner, 1988; Tulving, 1985) and thus asked participants to indicate whether they remembered the icon (remember), merely knew that it occurred in the learning phase (know), or whether the icon was new by clicking on the corresponding button below the icon. The description for remember and know judgments closely followed the ones used by Gardiner (1988), translated into German. After the test phase, participants provided some demographic information and could specify reasons that might warrant the exclusion of their data from the analyses and give comments regarding the study. Finally, they were thanked and debriefed.

**Fig. 4:**
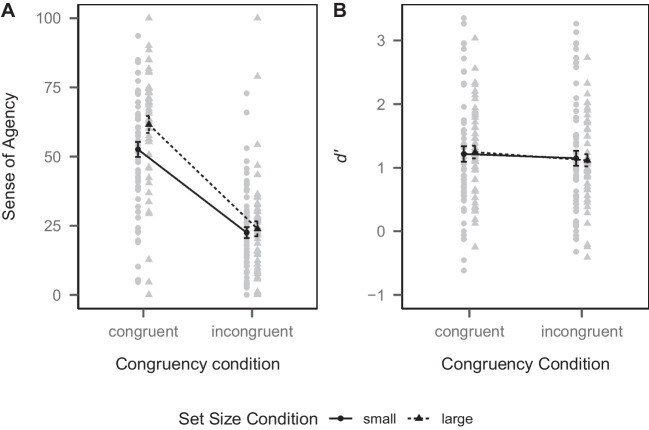
Mean sense of agency ratings (**A**) and mean *d’* values (**B**) by congruency and set size condition in Experiment 2. *Notes*. *Black shapes* depict group-level means, *gray shapes* depict person-level means. *Error bars* depict ± *SEM*

### Results

#### Sense of agency

Mean SoA ratings are depicted in Fig. 4A. To test whether SoA was higher in the congruent than in the incongruent condition (i.e., Hypothesis 1a), and whether this effect was modulated by set size condition (Hypothesis 1b), we performed a 2 (congruency condition) $$\times $$ 2 (set size condition) mixed-design ANOVA on the SoA ratings. There was a main effect of congruency condition ($$F(1, 109) = 311.10$$, $$ MSE = 201.95$$, $$p < .001$$, $$\hat{\eta }^2_p = .741$$, 95% CI [.66, .80]). SoA was higher in the congruent condition (*M* = 56.52, *SD* = 32.87) than in the incongruent condition (*M* = 23.09, *SD* = 25.61). Thus, Hypothesis 1a was supported. There was no main effect of set size condition ($$F(1, 109) = 2.66$$, $$ MSE = 552.72$$, $$p = .106$$, $$\hat{\eta }^2_p = .024$$, 95% CI [.00, .11]). SoA did not significantly differ between the small (*M* = 37.51, *SD* = 32.82) and large (*M* = 42.70, *SD* = 34.95) set condition. However, there was an interaction of congruency condition and set size condition ($$F(1, 109) = 3.94$$, $$ MSE = 201.95$$, $$p = .0496$$, $$\hat{\eta }^2_p = .035$$, 95% CI [.00, .13]). To test whether the interaction was as predicted, we computed the differences in mean SoA ratings between the congruent and incongruent condition for each participant and then performed an independent-samples *t*-test with the difference in mean SoA ratings as the dependent variable and set size condition as the independent variable (one-tailed testing). Indeed, the difference in SoA between the congruent and incongruent condition was larger in the large set condition than in the small set condition ($$\Delta M = 7.63$$, 95% CI $$[1.25, \infty ]$$, $$t(103.29) = 1.99$$, $$p = .025$$, $$\hat{d}$$ = 0.39, 95% CI [0.00, 0.78]). Thus, Hypothesis 1b was also supported.

#### Recognition memory

To test whether recognition memory was better for items in the congruent than in the incongruent condition (Hypothesis 2a), and whether this effect was modulated by set size condition (Hypothesis 2b), we collapsed across remember and know responses (treating both as “old” responses) and again computed the sensitivity index *d’* separately for the congruent and incongruent condition, as in Experiment 1. As there were again participants with extreme proportions of hit or false alarm rates, we again used the loglinear approach (Hautus, 1995) to adjust the rates, also as in Experiment 1. Mean *d’* values are depicted in Fig. 4B. We then performed a 2 (congruency condition) $$\times $$ 2 (set size condition) mixed-design ANOVA on *d’*. There was a main effect of congruency condition ($$F(1, 109) = 5.23$$, $$ MSE = 0.10$$, $$p = .024$$, $$\hat{\eta }^2_p = .046$$, 95% CI [.00, .14]). Recognition memory was better in the congruent condition (*M* = 1.23, *SD* = 0.84) than in the incongruent condition (*M* = 1.13, *SD* = 0.83), thus supporting Hypothesis 2a. There was no main effect of set size condition ($$F(1, 109) = 0.00$$, $$ MSE = 1.31$$, $$p = .989$$, $$\hat{\eta }^2_p = .000$$, 95% CI [.00, .00]). Recognition memory did not significantly differ between the small (*M* = 1.18, *SD* = 0.94) and large (*M* = 1.18, *SD* = 0.69) set condition. Critically, there was also no interaction between congruency condition and set size condition ($$F(1, 109) = 0.51$$, $$ MSE = 0.10$$, $$p = .475$$, $$\hat{\eta }^2_p = .005$$, 95% CI [.00, .06]). Thus, Hypothesis 2b was not supported.

**Table 1:** *M* (*SD)* of the proportion of remember and know judgments in each set size condition for old items in each congruency condition and for new items during the recognition test in Experiment 2

	Small set condition		Large set condition	
	Remember	Know	Remember	Know
Congruent condition	.35 (.21)	.30 (.15)	.38 (.21)	.28 (.14)
Incongruent condition	.34 (.21)	.30 (.16)	.34 (.20)	.29 (.15)
New items	.11 (.10)	.19 (.13)	.12 (.10)	.14 (.10)

**Fig. 5:**
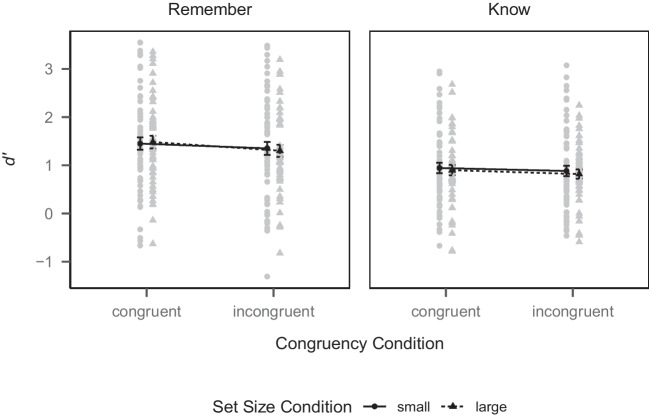
Mean *d’* values by congruency and set size condition for the remember and know subsets in Experiment 2. *Notes*. *Black shapes* depict group-level means, *gray shapes* depict person-level means. *Error bars* depict ± *SEM*

#### Remember/know distinction

Proportions of remember and know judgments in each set size condition for old items in each congruency condition and for new items are shown in Table 1. To test whether the set size conditions had a stronger influence on recollection than familiarity (Hypothesis 3), we split the data into two subsets. For the remember subset, we filtered trials where participants gave either a remember or new judgment (i.e., remember judgments for old items were considered hits, remember judgments for new items were considered false alarms, new judgments for old items were considered misses, and new judgments for new items were considered correct rejections). For the know subset, we filtered trials where participants gave either a know or a new judgment. We then computed *d’* separately for the congruent and incongruent condition for each subset of the data. Again, hit and false alarm rates needed to be adjusted. This time, however, within the subsets, the ratio of signal-to-noise trials could vary across participants. We thus first computed the ratio of signal-to-noise trials for each participant within the two subsets for each participant and then adjusted the hit and false alarm rates accordingly.^5^*d’* values for the remember and know subset are depicted in Fig. 5. We then performed 2 (congruency condition) $\times$ 2 (set size condition) mixed-design ANOVAs on *d’*, separately for the remember and know subset.

In the remember subset, there was a main effect of congruency condition ($$F(1, 109) = 6.11$$, $$ MSE = 0.17$$, $$p = .015$$, $$\hat{\eta }^2_p = .053$$, 95% CI [.00, .15]). Recognition memory was better in the congruent condition (*M* = 1.46, *SD* = 0.97) than in the incongruent condition (*M* = 1.33, *SD* = 0.98). There was no main effect of set size condition ($$F(1, 109) = 0.00$$, $$ MSE = 1.75$$, $$p = .961$$, $$\hat{\eta }^2_p = .000$$, 95% CI [.00, .00]). Recognition memory did not significantly differ between the small (*M* = 1.40, *SD* = 1.03) and large (*M* = 1.39, *SD* = 0.90) set condition. There was also no interaction between congruency condition and set size condition ($$F(1, 109) = 0.53$$, $$ MSE = 0.17$$, $$p = .469$$, $$\hat{\eta }^2_p = .005$$, 95% CI [.00, .06]).

In the know subset, there was no main effect of congruency condition ($$F(1, 109) = 1.96$$, $$ MSE = 0.14$$, $$p = .164$$, $$\hat{\eta }^2_p = .018$$, 95% CI [.00, .09]). Recognition memory did not significantly differ between the congruent condition (*M* = 0.92, *SD* = 0.81) and the incongruent condition (*M* = 0.85, *SD* = 0.78). There was also no main effect of set size condition ($$F(1, 109) = 0.15$$, $$ MSE = 1.14$$, $$p = .699$$, $$\hat{\eta }^2_p = .001$$, 95% CI [.00, .05]). Recognition memory did not significantly differ between the small (*M* = 0.91, *SD* = 0.86) and large (*M* = 0.86, *SD* = 0.71) set condition. Finally, there was also no interaction between congruency condition and set size condition ($$F(1, 109) = 0.03$$, $$ MSE = 0.14$$, $$p = .853$$, $$\hat{\eta }^2_p = .000$$, 95% CI [.00, .03]). Thus, set size condition neither had an effect in the remember nor in the know subset and therefore Hypothesis 3 was not supported. However, the effect of congruency condition was only present in the remember subset, but not in the know subset.

### Discussion

In Experiment 2, we again found a positive effect of action-effect congruency on the SoA, supporting Hypothesis 1a. We were thus able to replicate the effect from Experiment 1, which is also in line with earlier findings (Farrer et al., 2008; Hon & Yeo, 2021; Liesner et al., 2020; Tsuji & Imaizumi, 2022; Wegner & Wheatley, 1999). We further extended on this finding by showing that this effect is modulated by the size of the available action space. If participants were able to choose between more actions, the effect of action-effect congruency on the SoA was more pronounced, supporting Hypothesis 1b. This extends previous findings of higher SoA given a larger action space (Barlas et al., 2017; Barlas & Kopp, 2018; Barlas & Obhi, 2013). In this study, we found that a larger action space does not generally lead to a higher SoA, as we did not find a main effect, but that it interacts with action-effect congruency, yielding the highest SoA if the action space is larger and the environmental effect is congruent to the action.

We further again found a positive effect of action-effect congruency on recognition memory, supporting Hypothesis 2a and replicating the effect from Experiment 1 and Hon and Yeo (2021), using a different experimental paradigm and a less-controlled online setting. Thus, albeit being small, the effect appears to be quite robust. However, we did not find this effect to be modulated by the size of the available action space and thus, Hypothesis 2b was not supported. As in Experiment 1, we were confronted with the finding that a factor influencing SoA does not yield corresponding effects on recognition memory. This further supports the assumption that the effect of action-effect congruency on recognition memory may not be mediated by SoA, but that action-effect congruency may independently affect both SoA and recognition memory instead.

When we distinguished between two processes contributing to recognition memory performance, namely recollection and familiarity (Yonelinas, 2002), we did not find differential effects of the size of the available action space on the two processes. The number of available action alternatives did not have an effect when focusing on remember judgments (thought to reflect recollection), nor when focusing on know judgments (thought to reflect familiarity). Thus, Hypothesis 3 was not supported and the size of the available action space does not appear to affect recognition memory. However, we did find the effect of action-effect congruency to differ for recollection and familiarity, only being present in the case of recollection. This finding is inconsistent with Tsuji and Imaizumi (2022), who found no effect of action-effect congruency for both recollection and familiarity. Our findings on the other hand suggest that the effect of action-effect congruency on recognition memory is primarily driven by recollection.

## General discussion

In two experiments, we tested how congruency between an action and ensuing perceptual changes in the environment shape the sense of agency (SoA) and memory for the produced event, and how these effects are modulated by the predictability of the action effect and the size of the available action space (for an additional investigation of the effect of transient changes in control see Appendix A). In Experiment 1, participants moved a box by pressing one of two arrow keys and the box moved either congruently in the direction of the key press, or incongruently in the opposite direction. After the box stopped moving, a word appeared superimposed on the box, and memory for these words, together with novel words, was tested in a later old/new recognition test. Trials were either ordered randomly or in a blocked manner, such that all congruent and all incongruent trials occurred consecutively. In Experiment 2, participants colored an icon by selecting one of three or six colors, depending on their assigned condition. The icon was then colored congruently, in the selected color, or incongruently, in a color randomly chosen from the non-selected ones. Memory for these icons, together with novel icons, was tested in a later recognition test employing the remember/know procedure (Gardiner, 1988; Tulving, 1985). We consistently found action-effect congruency to increase the SoA (supporting Hypothesis 1/1a) and enhance recognition memory (supporting Hypothesis 2/2a), primarily recollection. We further found the predictability of the action effect to increase the SoA but not enhance recognition memory. Furthermore, the size of the available action space (i.e., the number of actions an agent can choose from) interacted with action-effect congruency, producing the highest SoA for effects congruent to an action selected from a larger set of possible responses. However, the size of the available action space did not affect recognition memory (not supporting Hypothesis 3 in Experiment 2).

### Action-effect congruency and effect predictability increase the sense of agency

We found the SoA to be higher if a perceptual effect following an agent’s action was congruent to that action. This was the case for both spatial congruency, where participants could move a box upwards or downwards with the box moving either in the specified or in the opposite direction (Experiment 1), and color congruency, where an image was either colored according to the agent’s color selection or in a different color (Experiment 2). This finding is very robust and has been reliably found in previous studies (Farrer et al., 2008; Hon & Yeo, 2021; Liesner et al., 2020; Tsuji & Imaizumi, 2022; Wegner & Wheatley, 1999). The influence of congruency between an action and ensuing effects in the environment may be explained by agents employing explicit reasoning processes to determine whether their action was causally responsible for the environmental effect (Liesner et al., 2020; Synofzik et al., 2008; Wegner, 2003) and may thus primarily affect the postdictive component of the SoA (see also Ebert & Wegner, 2010).

Further, we found the SoA to be higher if the action effect was predictable than if it was unpredictable in Experiment 1. Predictability was manipulated by making the effect (the box movement) always congruent or incongruent in a given experimental block compared to varying congruency on a trial-by-trial basis. This finding is consistent with Liesner et al. (2020) and suggests that the SoA is also affected by predictive components, such that agents predict their actions’ environmental effects (Blakemore & Frith, 2003; Frith, 2005; Haggard, 2017; Niziolek et al., 2013; Verschoor & Hommel, 2017), with this process being supported by the consistency of the occurrence of certain environmental effects. Action-effect congruency and predictability of the action effect increased SoA in a statistically independent manner, which suggests that postdictive and predictive processes shaping SoA operate in an independent manner.

### The size of the available action space modulates the effect of action-effect congruency on the sense of agency

In Experiment 2, we further found the effect of action-effect congruency on the SoA to be modulated by the size of the available action space, that being the number of actions an agent can choose from. The effect of action-effect congruency was more pronounced given a larger action space. While previous studies found a larger action space to increase the SoA (Barlas et al., 2017; Barlas & Kopp, 2018; Barlas & Obhi, 2013), our findings qualify these previous results by showing that this is not generally the case, but that this effect depends on (sufficient) congruency between an action and a subsequently ensuing effect. A possible explanation for this interaction is the predictability of the effect in incongruent action-effect episodes. With a smaller action space, the probability of one of the non-selected effects to occur by chance is higher than given a larger action space (e.g., 50% given three response options vs. 20% given six response options). The predictability of a given effect in incongruent trials may therefore be considered to be relatively higher for smaller compared to larger action spaces. Predictability increases the SoA (Liesner et al., 2020; Ma et al., 2019), which is also reflected in a higher SoA in the blocked order condition of Experiment 1, in which effects were perfectly predictable. Predictability would therefore decrease the difference in SoA between smaller and larger action spaces for incongruent trials. In addition, we generalize previous findings made with an implicit measure of agency (i.e., temporal binding), which may be a problematic measure of the SoA (Gutzeit et al., 2023) and may not be particularly related to explicit SoA ratings (Dewey & Knoblich, 2014; Saito et al., 2015; Schwarz et al., 2019). Here, we show an effect of the size of the available action space using explicit ratings of the SoA, which is in line with the congruence of explicit and implicit measures found by Barlas et al. (2017) and Barlas and Kopp (2018). Our findings suggest that a SoA only substantially emerges for effects that are congruent to an action and that these congruent effects stand out more in larger action spaces. Given that the SoA is higher for freely chosen compared to instructed actions (Barlas et al., 2017; Barlas & Obhi, 2013; Sebanz & Lackner, 2007; Sidarus et al., 2017; Villa et al., 2021), a larger available action space provides even more freedom of choice and may therefore enhance participants’ experience of control, thus increasing their SoA. The results are thus in line with accounts suggesting that greater involvement in action selection facilitates the SoA (Caspar et al., 2016; Haggard, 2008; Sebanz & Lackner, 2007).

### Action-effect congruency enhances recognition memory

Beyond its effect on the SoA, we found action-effect congruency to enhance recognition memory. Participants were better at recognizing words that were associated with a congruent effect than words associated with an incongruent effect. We found this influence in both experiments, using two different paradigms and both a laboratory and an online setting. This suggests that the influence is rather reliable, albeit being small. This is in line with findings by Hon and Yeo (2021), although our observed statistical effect sizes were smaller, but not in line with Tsuji and Imaizumi (2022), who found no such effect. However, a major difference in the study by Tsuji and Imaizumi (2022) is that it involved a baseline condition, in which the effect was unrelated to participants’ actions. This may have reduced participants’ perceptions of the contingency between their actions and subsequent effects. As we observed rather small effects, it is also possible that the study by Tsuji and Imaizumi (2022) was simply not sufficiently powered to detect effects of this size.

**Fig. 6:**
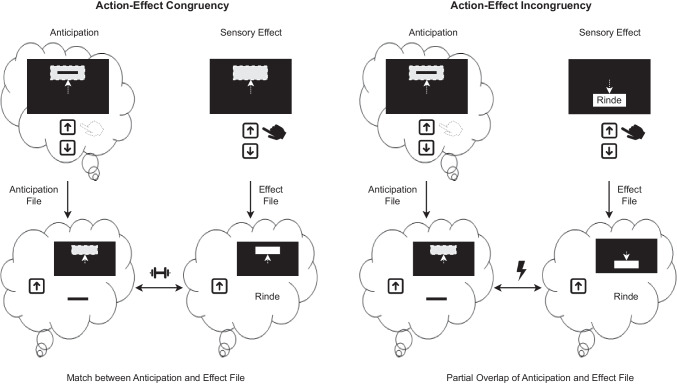
Depiction of the proposed feature-binding-based account for explaining the facilitating effect of action-effect congruency on recognition memory exemplary for the experimental paradigm used in Experiment 1. When planning or executing an action, agents anticipate the ensuing environmental effect of that action and store features related to the anticipated action event in an event file (anticipation file). After executing the action and receiving sensory feedback about the effect, agents store the features related to the actual action event in another event file (effect file). If the effect is congruent to the action, the anticipation and effect file match, and bindings between event features are strengthened. If the effect is incongruent to the action, the anticipation and effect file only partially overlap, causing interference

In Experiment 2, we further found the effect of action-effect congruency on recognition memory to primarily rely on the process of recollection, that is, the conscious and detailed memory of previously encountered events, including both the items and the context involved in the event (Yonelinas, 2002). This finding is inconsistent with an assumption that incongruent action-effect episodes might result in higher recollection due to expectancy violations, which could, for example, draw increased attention. Instead, the finding may point towards one possible explanation of the effect of action-effect congruency on memory, namely that stimuli associated with a congruent effect are perceived as self-generated and therefore as more self-relevant than stimuli associated with an incongruent effect, allowing them to be integrated into a broader network of self-relevant information. Indeed, effects of self-reference are mainly found in recollection (Conway & Dewhurst, 1995; van den Bos et al., 2010) and self-generated stimuli are better remembered than other-generated ones (Jacoby, 1978; Slamecka & Graf, 1978). In addition, if objects are assigned to the self, they are better discriminated from ones assigned to others (Kim & Johnson, 2012). This explanation suggests a close relationship of recognition memory and the SoA. For example, the opportunity to choose, which is associated with a higher SoA (Barlas et al., 2017; Barlas & Obhi, 2013; Sebanz & Lackner, 2007; Sidarus et al., 2017; Villa et al., 2021), has been found to also enhance declarative memory (Murty et al., 2015).

However, in the current research, we did not observe such a close correspondence between the SoA and recognition memory. While action-effect congruency led to both higher SoA and recognition memory, other factors increasing the SoA (the predictability of the action effect and the size of the available action space) did not affect recognition memory. This may suggest an at least partial dissociation of the SoA and recognition memory, with the SoA appearing to at least not fully mediate the effect of action-effect congruency on recognition memory. Rather, action-effect congruency may independently affect the SoA and memory. We thus deem the self-reference account to not be the most likely explanation for the relation between action-effect congruency and recognition memory. It should be noted however, that given the rather small effect of action-effect congruency on memory, further modulations of this effect, such as a scaling of the effect with SoA, may be difficult to detect.

Another possibility is that agents engage in effect anticipation, with the anticipated action-effect relationship being reinforced in the case of action-effect congruency. Upon planning or executing an action, agents may anticipate an ensuing sensory effect. This is proposed by both ideomotor theory (e.g., Greenwald, 1970; Hommel et al., 2001) and predictive processing theories (e.g., Friston et al., 2016; Friston et al., 2017; Seth, 2014). For example, these predictions are reflected in anticipatory eye movements towards predicted effect locations (Pfeuffer et al., 2016). Importantly, these predictions, as do the actual action and effect, involve the binding of various features associated with the action event (Frings et al., 2020; Hommel et al., 2001). According to the theory of event coding (Hommel et al., 2001), environmental events are represented by feature codes, with feature codes referring to the same event getting bound together in an “event file” (Hommel, 1998, 2004). This event file thus includes feature codes describing various aspects of the event, such as the action itself, perceptual effects produced by the action, or stimuli involved in the event (Frings et al., 2020). There is ample evidence for such bindings between action and effect features (e.g., Dutzi & Hommel, 2009; Elsner & Hommel, 2001; Moeller et al., 2016, 2019). Agents may therefore first create an initial anticipatory event file (anticipation file) and, after execution of the action and observation of the ensuing effect, create a second event file (effect file, see Fig. 6). In the case of action-effect congruency, this second effect file matches the anticipation file, reinforcing the bindings between feature codes in the anticipation file and potentially updating it with new information (e.g., the word as a part of the effect in Experiment 1, which could not be anticipated). The match produced by the two event files may also drive the predictive component of the SoA (Blakemore & Frith, 2003; Frith, 2005; Haggard, 2017; Niziolek et al., 2013; Verschoor & Hommel, 2017). In the case of action-effect incongruency, the effect file only partially overlaps with the anticipation file, causing interference. Such interference between event files is evident, for example, in partial repetition costs, being reflected in delayed and more error-prone responses if features of an intervening event only partially overlap with features of an event held in memory compared to when these features do not overlap or completely overlap (e.g., Fournier et al., 2022; Hommel, 1998; Mocke et al., 2023). The finding that the effect of action-effect congruency primarily relies on recollection, which involves retrieval of contextual features of an event, is in line with this reasoning, suggesting that the integration of different event features is necessary for the effect to emerge. Thus, the absence of interference between the anticipation and effect file and the stronger integration of features of an event into a common event file may explain why action-effect congruency enhances recognition memory, as it might allow those features to serve as retrieval cues.

Yet another possible explanation, which may also contribute to the other potential explanations discussed, includes modulations in attention allocation. Agents allocate more attention to stimuli under their control (Huffman & Brockmole, 2020; Wen & Haggard, 2018) and stimuli receiving more attention are recognized better (Craik et al., 1996; Naveh-Benjamin et al., 1998). Anticipatory eye movements towards predicted effects (Pfeuffer et al., 2016) in Experiment 1 may also give stimuli appearing in the expected location a slight processing advantage during encoding. Similar mechanisms may be at play in Experiment 2. For example, color perception in the periphery of the visual field is limited (Hansen et al., 2009) and is thus probably worse if stimuli are not fixated than when they are fixated. Anticipatory eye movements may lead to faster fixation and thus to a better color perception. These factors may contribute to the facilitating effect of action-effect congruency on recognition memory.

## Conclusion

To conclude, we investigated the effect of congruency between an agent’s action and an ensuing effect in the agent’s environment on the SoA and recognition memory, as well as factors that may modulate these effects, namely the predictability of the environmental effect and the size of the action space the agent can select an action from. In terms of the SoA, we found action-effect congruency to increase the SoA, consistent with many previous findings. We further found this effect to be increased when the available action space was larger and environmental effects were predictable. Together, these results suggest the joint, possibly independent, additive influence of predictive and postdictive processes on the SoA. In terms of memory, we found action-effect congruency to enhance recognition memory, primarily affecting the process of recollection. However, the predictability of the environmental effect and the size of the available action space did not affect recognition memory. This partial dissociation of factors influencing the SoA and recognition memory cast doubt on the assumption that the effect of action-effect congruency on recognition memory is mediated by the SoA, although further modulations of this effect may be difficult to detect given the rather small effect sizes observed. Instead, action-effect congruency may influence the two constructs independently. The effect of action-effect congruency on recognition memory could be explained by accounts of self-referential processing, feature binding, and attention allocation.

## Data Availability

All data, analysis code, and research materials have been made publicly available at the Open Science Framework (OSF) and are accessible at https://osf.io/5r3ms/. All experiments were preregistered (Experiment 1: https://doi.org/10.17605/OSF.IO/SF6JE, Experiment 2: https://doi.org/10.17605/OSF.IO/MBXK4).

## Appendix A: Change in control

### Experiment 1

In Experiment 1, we additionally investigated whether the effect of action-effect congruency on recognition memory is sensitive to changes in control. In the condition in which action-effect congruency varied from trial to trial (random order condition), a transition from a congruent to an incongruent trial and vice versa would be associated with a change in control, whereas the repetition of a congruent or incongruent trial would not. Remembering stimuli associated with changes in control may be particularly important, as these may signal contexts one wishes to approach or avoid in the future. In that sense, memory sensitivity to changes in control may be adaptive. We therefore expected that recognition memory is better if the trial in which a stimulus occurred is associated with a change in control than if it is associated with no change in control compared to the previous trial (Hypothesis 4). We further considered possible differential effects of a gain in control (transition from an incongruent to a congruent trial) and loss of control (transition from a congruent to an incongruent trial) as an exploratory question. For example, it has been found that low SoA in a preceding trial is associated with increased inhibitory control in a subsequent trial (Ren et al., 2023). An up-regulation of inhibitory control could occur immediately after detecting a loss in control, which may benefit the encoding of the effect content in the same trial. Alternatively, it could occur in the subsequent trial and may add to possibly beneficial effects of high control on memory.

#### Results

To test if recognition memory was better for words occurring in trials associated with a change in control from the previous trial compared to trials associated with no change in control (i.e., Hypothesis 4), we fit a generalized mixed linear model with a logit link function to the data from the random order condition, with hits (1 = hit, 0 = miss) as the binary dependent variable and contrast-coded change in control as the independent variable, as well as random person intercepts. We tested planned contrasts reflecting the difference between change in control and no change in control, and between a gain and a loss in control. Words occurring in the first learning trial were omitted, as no change in control could occur in the first trial. The planned contrasts revealed that there was no difference in recognition memory between trials associated with a change in control (*M* = 0.59, *SD* = 0.49) and trials associated with no change in control (*M* = 0.59, *SD* = 0.49, log OR = $0.00$, 95% CI $[-0.06, 0.06]$, $z = 0.02$, $p = .986$)^6^, or between trials associated with a gain in control (*M* = 0.61, *SD* = 0.49) and trials associated with a loss in control (*M* = 0.58, *SD* = 0.49, log OR = $0.06$, 95% CI $[-0.06, 0.19]$, $z = 1.01$, $p = .314$). Thus, Hypothesis 4 was not supported.

As a supplemental analysis, we fit the model with the grand-mean-centered difference in SoA from the associated trial and the previous trial in the learning phase as the independent variable, investigating both a linear and a quadratic trend. Neither the linear trend ($\hat{b} = 0.13$, 95% CI $[-0.07, 0.34]$, $z = 1.27$, $p = .205$) nor the quadratic trend ($\hat{b} = -0.11$, 95% CI $[-0.44, 0.22]$, $z = -0.64$, $p = .520$) were significant, suggesting no trial-to-trial modulation of recognition memory by changes in SoA.

### Experiment 2

In Experiment 2, we investigated the influence of a change in control in an exploratory analysis.

#### Results

The analysis was equivalent to the one in Experiment 1. The contrast analysis revealed that there was no difference in recognition memory between trials associated with a change in control (*M* = 0.62, *SD* = 0.49) and trials associated with no change in control (*M* = 0.63, *SD* = 0.48, log OR = $-0.02$, 95% CI $[-0.05, 0.02]$, $z = -0.94$, $p = .346$). However, there was a significant difference between trials associated with a gain in control and trials associated with a loss in control. Recognition memory was better for trials associated with a gain in control (*M* = 0.65, *SD* = 0.48) than for trials associated with a loss in control (*M* = 0.59, *SD* = 0.49, log OR = $0.13$, 95% CI $[0.04, 0.22]$, $z = 2.81$, $p = .005$).

### Discussion

We found mixed evidence for effects of transient changes in control on recognition memory. While a general comparison of memory performance in trials associated with a change in control compared to trials associated with no change in control yielded no evidence for an effect of change in control in both experiments (not supporting Hypothesis 4 in Experiment 1), we found recognition memory to be enhanced for trials associated with a gain in control compared to trials associated with a loss in control in the exploratory analysis in Experiment 2, but not in the exploratory analysis in Experiment 1. Thus, participants’ memory in Experiment 1 may not have been sensitive to transient changes in control. However, a possible reason for the discrepancy between the experiments is that we could only use the data of one between-subjects condition (the random order condition) for this test in Experiment 1, whereas we could use all the data in Experiment 2 for this analysis, resulting in a higher power in Experiment 2. Therefore, the effect may have been too small to be reliably detectable in Experiment 1, but at least descriptively it went in the same direction as in Experiment 2.

Changes in control can modulate attention (Wen & Haggard, 2018) and it has previously been found that reduced control in a previous trial increases inhibitory control in a subsequent one (Ren et al., 2023). Thus, participants may allocate more attention to, and be able to better inhibit distracting information in, trials associated with a gain in control. However, this effect was not consistently observed and should be further investigated in future research. What may limit the effect, particularly the overall effect of change in control, is the possibility of opposing influences on memory performance. For example, a memory advantage due to high control in the current trial may compensate for a missing increase in inhibitory control from the previous trial.

## Appendix B: Effects of block order in Experiment 1

The order of the blocks in the blocked order condition of Experiment 1 was counterbalanced, such that approximately half the participants in this condition were first confronted with congruent trials only and the other half were first confronted with incongruent trials only. As an exploratory analysis, we tested whether the ordering of the two blocks affected the SoA and recognition memory. For these analyses, we only considered data from the blocked order condition.

For testing the effect of block order on SoA, we performed a 2 (congruency condition) $$\times $$ 2 (block order) mixed-design ANOVA on the SoA ratings. While the main effect of congruency condition was significant ($$F(1, 59) = 64.94$$, $$ MSE = 0.06$$, $$p < .001$$, $$\hat{\eta }^2_p = .524$$, 95% CI [.35, .65]), with higher SoA ratings in the congruent condition (*M* = 0.89, *SD* = 0.16) than in the incongruent condition (*M* = 0.53, *SD* = 0.35), there was neither a main effect of block order (congruent first: *M* = 0.76, *SD* = 0.30, incongruent first: *M* = 0.66, *SD* = 0.35, $$F(1, 59) = 3.75$$, $$ MSE = 0.09$$, $$p = .057$$, $$\hat{\eta }^2_p = .060$$, 95% CI [.00, .21]), nor an interaction ($$F(1, 59) = 1.05$$, $$ MSE = 0.06$$, $$p = .309$$, $$\hat{\eta }^2_p = .018$$, 95% CI [.00, .13]). This suggests that the block order did not affect the SoA.

For testing the effect of block order on recognition memory, we performed a 2 (congruency condition) $$\times $$ 2 (block order) mixed-design ANOVA on *d’*. While there were neither a main effect of congruency condition (congruent condition: *M* = 0.81, *SD* = 0.79, incongruent condition: *M* = 0.74, *SD* = 0.77, $$F(1, 59) = 1.20$$, $$ MSE = 0.12$$, $$p = .278$$, $$\hat{\eta }^2_p = .020$$), nor a main effect of block order (congruent first: *M* = 0.81, *SD* = 0.72, incongruent first: *M* = 0.74, *SD* = 0.84, $$F(1, 59) = 1.20$$, $$ MSE = 0.12$$, $$p = .278$$, $$\hat{\eta }^2_p = .020$$), there was a significant interaction ($$F(1, 59) = 18.13$$, $$ MSE = 0.12$$, $$p < .001$$, $$\hat{\eta }^2_p = .235$$).^7^ Post-hoc pairwise comparisons revealed that recognition memory was better in the condition that was presented first. In detail, congruent trials were remembered better than incongruent ones when congruent trials were presented first ($$\Delta M = 0.33$$, 95% CI $$[0.16, 0.50]$$, $$t(59) = 3.82$$, $$p < .001$$, $$\hat{d}$$ = 0.50, 95% CI [0.22, 0.77]) and inversely, incongruent trials were remembered better when they were presented first ($$\Delta M = -0.20$$, 95% CI $$[-0.37, -0.02]$$, $$t(59) = -2.22$$, $$p = .030$$, $$\hat{d}$$ = -0.29, 95% CI [-0.55, -0.03]). This may suggest the presence of primacy effects.

To test for primacy effects and whether they may be responsible for the non-significant findings in the blocked order condition, we added trial position in the learning phase of the tested word (divided by 80 to recode to the interval [0, 1]) as a continuous trial-level covariate to the generalized mixed linear model (using the whole data) with hits (1 = hit, 0 = miss) as the binary dependent variable. Planned contrasts testing the difference between the congruent and incongruent condition within the random and blocked order conditions served as the independent variables. The model further included random person intercepts. Trial position was indeed a significant predictor (log OR = $-0.43$, 95% CI $[-0.65, -0.21]$, $z = -3.91$, $p < .001$), further solidifying the presence of primacy effects. However, recognition performance within the blocked order condition was still not significantly better in the congruent than in the incongruent condition (log OR = $0.06$, 95% CI $[-0.03, 0.15]$, $z = 1.39$, $p = .165$), whereas this was the case within the random order condition (log OR = $0.09$, 95% CI $[0.00, 0.18]$, $z = 2.04$, $p = .041$).^8^ Thus, controlling for primacy effects did not change the results.

## Footnotes

1 Due to an experimenter error, one participant was accidentally assigned to the blocked instead of the random order condition. Thirty-one participants in the blocked order condition first conducted the congruent block and 30 participants first conducted the incongruent block.

2 This adjustment was not preregistered in Experiment 1.

3 As we only computed one false alarm rate per participant, which we contrasted with the two hit rates from the experimental conditions, a possible concern is that this may induce dependence between the *d’* values for the two conditions. To address this concern, we calculated bootstrapped estimates of the false alarm rates: We randomly assigned half of the new words to the congruent condition and half to the incongruent condition a total of 1000 times and computed the respective false alarm frequencies and rates per participant and condition. We then used the means of the resulting distributions as estimates for calculating *d’* (for the adjustment we consequently added 0.5 to both the number of hits and false alarms as the ratio of signal-to-noise trials was now balanced). This did not substantially affect the results of both Experiment 1 and 2, with the exception that the contrast comparing the congruent and incongruent condition within the random order condition of Experiment 1 reached significance (*p* = .048). Therefore, a possible non-independence of *d’* values from different conditions as a result of calculating them using the same person-specific false alarm rate does not appear to be a problem.

4 With these proportions we aimed to make the exclusion criterion comparable between conditions with different numbers of response options. They are also roughly proportional to the ones used in Experiment 1, which only included two response options.

5 For example, if there were 20 signal and 30 noise trial in the subset for a given participant, we added 0.4 to the number of hits (for both the congruent and incongruent condition) and 0.6 to the number of false alarms, 0.8 to the number of signal trials and 1.2 to the number of noise trials. These values therefore reflect the proportion of signal-to-noise trials for this participant but could vary across participants.

## References

[CR1] Aust, F., & Barth, M. (2023). *papaja: Prepare reproducible APA journal articles with R Markdown. R package version 0.1.2*. https://github.com/crsh/papaja

[CR2] Barlas, Z., & Kopp, S. (2018). Action choice and outcome congruency independently affect intentional binding and feeling of control judgments. *Frontiers in Human Neuroscience,**12*. 10.3389/fnhum.2018.00137 10.3389/fnhum.2018.00137 PMC5904194 29695958

[CR3] Barlas, Z., & Obhi, S. (2013). Freedom, choice, and the sense of agency. *Frontiers in Human Neuroscience,**7*. 10.3389/fnhum.2013.00514 10.3389/fnhum.2013.00514 PMC3756740 24009575

[CR4] Barlas, Z., Hockley, W. E., & Obhi, S. S. (2017). The effects of freedom of choice in action selection on perceived mental effort and the sense of agency. *Acta Psychologica,**180*, 122–129. 10.1016/j.actpsy.2017.09.004 28942124 10.1016/j.actpsy.2017.09.004

[CR5] Barth, M. (2023). *tinylabels: Lightweight variable labels. R package version 0.2.4*. https://cran.r-project.org/package=tinylabels

[CR6] Bates, D. M., Mächler, M., Bolker, B. M., & Walker, S. (2015). Fitting linear mixed-effects models using lme4. *Journal of Statistical Software*, *67*(1), 1–48. 10.18637/jss.v067.i01

[CR7] Beck, B., Di Costa, S., & Haggard, P. (2017). Having control over the external world increases the implicit sense of agency. *Cognition,**162*, 54–60. 10.1016/j.cognition.2017.02.002 28212896 10.1016/j.cognition.2017.02.002

[CR8] Ben-Shachar, M. S., Lüdecke, D., & Makowski, D. (2020). effectsize: Estimation of effect size indices and standardized parameters. *Journal of Open Source Software,**5*(56), 2815. 10.21105/joss.02815

[CR9] Blakemore, S.-J., & Frith, C. (2003). Self-awareness and action. *Current Opinion in Neurobiology,**13*(2), 219–224. 10.1016/S0959-4388(03)00043-6 12744977 10.1016/s0959-4388(03)00043-6

[CR10] Borhani, K., Beck, B., & Haggard, P. (2017). Choosing, doing, and controlling: Implicit sense of agency over somatosensory events. *Psychological Science,**28*(7), 882–893. 10.1177/0956797617697693 28488908 10.1177/0956797617697693

[CR11] Caspar, E. A., Christensen, J. F., Cleeremans, A., & Haggard, P. (2016). Coercion changes the sense of agency in the human brain. *Current Biology,**26*(5), 585–592. 10.1016/j.cub.2015.12.067 26898470 10.1016/j.cub.2015.12.067 PMC4791480

[CR12] Chambon, V., Sidarus, N., & Haggard, P. (2014). From action intentions to action effects: How does the sense of agency come about? *Frontiers in Human Neuroscience,**8*, 320. 10.3389/fnhum.2014.00320 24860486 10.3389/fnhum.2014.00320 PMC4030148

[CR13] Chiu, Y.-C., & Egner, T. (2015). Inhibition-induced forgetting: When more control leads to less memory. *Psychological Science,**26*(1), 27–38. 10.1177/0956797614553945 25398560 10.1177/0956797614553945 PMC4353579

[CR14] Conway, M. A., & Dewhurst, S. A. (1995). The self and recollective experience. *Applied Cognitive Psychology,**9*(1), 1–19. 10.1002/acp.2350090102

[CR15] Craik, F. I. M., Govoni, R., Naveh-Benjamin, M., & Anderson, N. D. (1996). The effects of divided attention on encoding and retrieval processes in human memory. *Journal of Experimental Psychology: General,**125*(2), 159–180. 10.1037/0096-3445.125.2.159 8683192 10.1037//0096-3445.125.2.159

[CR16] Dewey, J. A., & Knoblich, G. (2014). Do implicit and explicit measures of the sense of agency measure the same thing? *PLoS One,**9*(10), e110118. 10.1371/journal.pone.0110118 25330184 10.1371/journal.pone.0110118 PMC4199671

[CR17] Dutzi, I. B., & Hommel, B. (2009). The microgenesis of action-effect binding. *Psychological Research,**73*(3), 425–435. 10.1007/s00426-008-0161-7 18810487 10.1007/s00426-008-0161-7

[CR18] Ebert, J. P., & Wegner, D. M. (2010). Time warp: Authorship shapes the perceived timing of actions and events. *Consciousness and Cognition,**19*(1), 481–489. 10.1016/j.concog.2009.10.002 19896868 10.1016/j.concog.2009.10.002 PMC2836403

[CR19] Elsner, B., & Hommel, B. (2001). Effect anticipation and action control. *Journal of Experimental Psychology: Human Perception and Performance,**27*(1), 229–240. 10.1037/0096-1523.27.1.229 11248937 10.1037//0096-1523.27.1.229

[CR20] Engelkamp, J. (1986). Nouns and verbs in paired-associate learning: Instructional effects. *Psychological Research,**48*(3), 153–159. 10.1007/BF00309163

[CR21] Farrer, C., Bouchereau, M., Jeannerod, M., & Franck, N. (2008). Effect of distorted visual feedback on the sense of agency. *Behavioural Neurology,**19*(1–2), 53–57. 10.1155/2008/425267 18413918 10.1155/2008/425267 PMC5452467

[CR22] Faul, F., Erdfelder, E., Buchner, A., & Lang, A.-G. (2009). Statistical power analyses using G*Power 3.1: Tests for correlation and regression analyses. *Behavior Research Methods,**41*(4), 1149–1160. 10.3758/BRM.41.4.1149 19897823 10.3758/BRM.41.4.1149

[CR23] Fournier, L. R., Richardson, B. P., & Logan, G. D. (2022). Partial repetition costs are reduced but not eliminated with practice. *Journal of Cognition,**5*(1), 37. 10.5334/joc.230 36072096 10.5334/joc.230 PMC9400617

[CR24] Frings, C., Hommel, B., Koch, I., Rothermund, K., Dignath, D., Giesen, C., ... Philipp, A. (2020). Binding and Retrieval in Action Control (BRAC). *Trends in Cognitive Sciences,**24*(5), 375–387. 10.1016/j.tics.2020.02.004 10.1016/j.tics.2020.02.004 32298623

[CR25] Friston, K., FitzGerald, T., Rigoli, F., Schwartenbeck, P., O’Doherty, J., & Pezzulo, G. (2016). Active inference and learning. *Neuroscience & Biobehavioral Reviews,**68*, 862–879. 10.1016/j.neubiorev.2016.06.022 27375276 10.1016/j.neubiorev.2016.06.022 PMC5167251

[CR26] Friston, K., FitzGerald, T., Rigoli, F., Schwartenbeck, P., & Pezzulo, G. (2017). Active inference: A process theory. *Neural Computation,**29*(1), 1–49. 10.1162/NECO_a_00912 27870614 10.1162/NECO_a_00912

[CR27] Frith, C. (2005). The self in action: Lessons from delusions of control. *Consciousness and Cognition,**14*(4), 752–770. 10.1016/j.concog.2005.04.002 16098765 10.1016/j.concog.2005.04.002

[CR28] Gardiner, J. M. (1988). Functional aspects of recollective experience. *Memory & Cognition,**16*(4), 309–313. 10.3758/BF03197041 3210971 10.3758/bf03197041

[CR29] Goldstein, H. (2011). *Multilevel statistical models* (4th ed.). Wiley.

[CR30] Green, P., & MacLeod, C. J. (2016). SIMR: An R package for power analysis of generalized linear mixed models by simulation. *Methods in Ecology and Evolution,**7*(4), 493–498. 10.1111/2041-210X.12504

[CR31] Greenwald, A. G. (1970). Sensory feedback mechanisms in performance control: With special reference to the ideo-motor mechanism. *Psychological Review,**77*(2), 73–99. 10.1037/h0028689 5454129 10.1037/h0028689

[CR32] Gutzeit, J., Weller, L., Kürten, J., & Huestegge, L. (2023). Intentional binding: Merely a procedural confound? *Journal of Experimental Psychology: Human Perception and Performance,**49*(6), 759–773. 10.1037/xhp0001110 37166936 10.1037/xhp0001110

[CR33] Haggard, P. (2008). Human volition: Towards a neuroscience of will. *Nature Reviews Neuroscience,**9*(12), 934–946. 10.1038/nrn2497 19020512 10.1038/nrn2497

[CR34] Haggard, P. (2017). Sense of agency in the human brain. *Nature Reviews Neuroscience,**18*, 196–207. 10.1038/nrn.2017.14 28251993 10.1038/nrn.2017.14

[CR35] Haggard, P., & Tsakiris, M. (2009). The experience of agency: Feelings, judgments, and responsibility. *Current Directions in Psychological Science,**18*(4), 242–246. 10.1111/j.1467-8721.2009.01644.x

[CR36] Hansen, T., Pracejus, L., & Gegenfurtner, K. R. (2009). Color perception in the intermediate periphery of the visual field. *Journal of Vision,**9*(4), 26. 10.1167/9.4.26 10.1167/9.4.26 19757935

[CR37] Hautus, M. J. (1995). Corrections for extreme proportions and their biasing effects on estimated values of d. *Behavior Research Methods, Instruments, & Computers,**27*(1), 46–51. 10.3758/bf03203619

[CR38] Henninger, F., Shevchenko, Y., Mertens, U. K., Kieslich, P. J., & Hilbig, B. E. (2022). lab.js: A free, open, online study builder. *Behavior Research Methods,**54*, 556–573. 10.3758/s13428-019-01283-5 34322854 10.3758/s13428-019-01283-5 PMC9046347

[CR39] Hoffman, L., & Rovine, M. J. (2007). Multilevel models for the experimental psychologist: Foundations and illustrative examples. *Behavior Research Methods,**39*(1), 101–117. 10.3758/BF03192848 17552476 10.3758/bf03192848

[CR40] Hommel, B. (1998). Event files: Evidence for automatic integration of stimulus-response episodes. *Visual Cognition,**5*(1–2), 183–216. 10.1080/713756773

[CR41] Hommel, B. (2004). Event files: Feature binding in and across perception and action. *Trends in Cognitive Sciences,**8*(11), 494–500. 10.1016/j.tics.2004.08.007 15491903 10.1016/j.tics.2004.08.007

[CR42] Hommel, B., Müsseler, J., Aschersleben, G., & Prinz, W. (2001). The Theory of Event Coding (TEC): A framework for perception and action planning. *Behavioral and Brain Sciences,**24*(5), 849–878. 10.1017/s0140525x01000103 12239891 10.1017/s0140525x01000103

[CR43] Hon, N., & Yeo, N. (2021). Having a sense of agency can improve memory. *Psychonomic Bulletin & Review,**28*(3), 946–952. 10.3758/s13423-020-01849-x 33415660 10.3758/s13423-020-01849-x

[CR44] Huffman, G., & Brockmole, J. R. (2020). Attentional selection is biased towards controllable stimuli. *Attention, Perception, & Psychophysics,**82*(5), 2558–2569. 10.3758/s13414-020-02004-3 10.3758/s13414-020-02004-3 32166643

[CR45] Jacoby, L. L. (1978). On interpreting the effects of repetition: Solving a problem versus remembering a solution. *Journal of Verbal Learning and Verbal Behavior,**17*(6), 649–667. 10.1016/S0022-5371(78)90393-6

[CR46] Jainta, B., Siestrup, S., El-Sourani, N., Trempler, I., Wurm, M. F., Werning, M., ... Schubotz, R. I. (2022). Seeing what i did (not): Cerebral and behavioral effects of agency and perspective on episodic memory re-activation. *Frontiers in Behavioral Neuroscience,**15*, 793115. 10.3389/fnbeh.2021.793115 10.3389/fnbeh.2021.793115 PMC8777223 35069141

[CR47] Kim, K., & Johnson, M. K. (2012). Extended self: Medial prefrontal activity during transient association of self and objects. *Social Cognitive and Affective Neuroscience,**7*(2), 199–207. 10.1093/scan/nsq096 21148177 10.1093/scan/nsq096 PMC3277366

[CR48] Kuznetsova, A., Brockhoff, P. B., & Christensen, R. H. B. (2017). lmerTest package: Tests in linear mixed effects models. *Journal of Statistical Software,**82*(13), 1–26. 10.18637/jss.v082.i13

[CR49] Lange, K., Kühn, S., & Filevich, E. (2015). “Just Another Tool for Online Studies’’ (JATOS): An easy solution for setup and management of web servers supporting online studies. *PLoS One,**10*(6), e0130834. 10.1371/journal.pone.0130834 26114751 10.1371/journal.pone.0130834 PMC4482716

[CR50] Lenth, R. (2023). *emmeans: Estimated marginal means, aka least-squares means. R package version 1.8.9.*[SPACE]https://cran.r-project.org/package=emmeans

[CR51] Liesner, M., Kirsch, W., & Kunde, W. (2020). The interplay of predictive and postdictive components of experienced selfhood. *Consciousness and Cognition,**77*, 102850. 10.1016/j.concog.2019.102850 31731032 10.1016/j.concog.2019.102850

[CR52] Ma, K., Hommel, B., & Chen, H. (2019). Context-induced contrast and assimilation effects in explicit and implicit measures of agency. *Scientific Reports,**9*(1), 3883. 10.1038/s41598-019-40545-2 30846800 10.1038/s41598-019-40545-2 PMC6405998

[CR53] Mathôt, S., Schreij, D., & Theeuwes, J. (2012). OpenSesame: An open-source, graphical experiment builder for the social sciences. *Behavior Research Methods,**44*(2), 314–324. 10.3758/s13428-011-0168-7 22083660 10.3758/s13428-011-0168-7 PMC3356517

[CR54] Mocke, V., Benini, E., Parmar, J., Schiltenwolf, M., & Kunde, W. (2023). What is behind partial repetition costs? Event-files do not fully occupy bound feature codes. *Psychonomic Bulletin & Review,**30*(4), 1463–1474. 10.3758/s13423-023-02253-x 36867367 10.3758/s13423-023-02253-x PMC10482800

[CR55] Moeller, B., Pfister, R., Kunde, W., & Frings, C. (2016). A common mechanism behind distractor-response and response-effect binding? *Attention, Perception, & Psychophysics,**78*(4), 1074–1086. 10.3758/s13414-016-1063-1 10.3758/s13414-016-1063-1 26810573

[CR56] Moeller, B., Pfister, R., Kunde, W., & Frings, C. (2019). Selective binding of stimulus, response, and effect features. *Psychonomic Bulletin & Review,**26*(5), 1627–1632. 10.3758/s13423-019-01646-1 31325038 10.3758/s13423-019-01646-1

[CR57] Murty, V. P., DuBrow, S., & Davachi, L. (2015). The simple act of choosing influences declarative memory. *The Journal of Neuroscience,**35*(16), 6255–6264. 10.1523/JNEUROSCI.4181-14.2015 25904779 10.1523/JNEUROSCI.4181-14.2015 PMC4405547

[CR58] Naefgen, C., Dambacher, M., & Janczyk, M. (2018). Why free choices take longer than forced choices: Evidence from response threshold manipulations. *Psychological Research,**82*(6), 1039–1052. 10.1007/s00426-017-0887-1 28776264 10.1007/s00426-017-0887-1

[CR59] Naveh-Benjamin, M., Craik, F. I. M., Guez, J., & Dori, H. (1998). Effects of divided attention on encoding and retrieval processes in human memory: Further support for an asymmetry. *Journal of Experimental Psychology: Learning, Memory, and Cognition,**24*(5), 1091–1104. 10.1037/0278-7393.24.5.1091 9747524 10.1037//0278-7393.24.5.1091

[CR60] Niziolek, C. A., Nagarajan, S. S., & Houde, J. F. (2013). What does motor efference copy represent? Evidence from speech production. *The Journal of Neuroscience,**33*(41), 16110–16116. 10.1523/JNEUROSCI.2137-13.2013 24107944 10.1523/JNEUROSCI.2137-13.2013 PMC3792453

[CR61] Pfeuffer, C. U., Kiesel, A., & Huestegge, L. (2016). A look into the future: Spontaneous anticipatory saccades reflect processes of anticipatory action control. *Journal of Experimental Psychology: General,**145*(11), 1530–1547. 10.1037/xge0000224 27797559 10.1037/xge0000224

[CR62] R Core Team. (2023). *R: A language and environment for statistical computing.* R Foundation for Statistical Computing.

[CR63] Raaijmakers, J. G. W., & Shiffrin, R. M. (1980). SAM: A theory of probabilistic search of associative memory. In G. H. Bower (Ed.), *Psychology of learning and motivation* (vol. 14, pp. 207–262). Academic Press. 10.1016/S0079-7421(08)60162-0

[CR64] Raaijmakers, J. G. W., & Shiffrin, R. M. (1981). Search of associative memory. *Psychological Review,**88*(2), 93–134. 10.1037/0033-295X.88.2.93

[CR65] Ramachandran, V. S., & Rogers-Ramachandran, D. (1997). Synaesthesia in phantom limbs induced with mirrors. *Proceedings of the Royal Society of London. Series B: Biological Sciences,**263*(1369), 377–386. 10.1098/rspb.1996.0058 10.1098/rspb.1996.0058 8637922

[CR66] Ren, Q., Kaiser, J., Gentsch, A., & Schütz-Bosbach, S. (2023). Prepared to stop: How sense of agency in a preceding trial modulates inhibitory control in the current trial. *Cerebral Cortex,**33*(13), 8565–8580. 10.1093/cercor/bhad141 37125462 10.1093/cercor/bhad141

[CR67] Roberts, B. R. T., MacLeod, C. M., & Fernandes, M. A. (2022). The enactment effect: A systematic review and meta-analysis of behavioral, neuroimaging, and patient studies. *Psychological Bulletin,**148*, 397–434. 10.1037/bul0000360 35878067 10.1037/bul0000360

[CR68] Ruiz, N. A., DuBrow, S., & Murty, V. P. (2023). Agency as a bridge to form associative memories. *Journal of Experimental Psychology: General.*[SPACE]10.1037/xge0001356 36862492 10.1037/xge0001356

[CR69] Saito, N., Takahata, K., Murai, T., & Takahashi, H. (2015). Discrepancy between explicit judgement of agency and implicit feeling of agency: Implications for sense of agency and its disorders. *Consciousness and Cognition,**37*, 1–7. 10.1016/j.concog.2015.07.011 26253893 10.1016/j.concog.2015.07.011

[CR70] Schwarz, K. A., Klaffehn, A. L., Hauke-Forman, N., Muth, F. V., & Pfister, R. (2022). Never run a changing system: Action-effect contingency shapes prospective agency. *Cognition,**229*, 105250. 10.1016/j.cognition.2022.105250 35963118 10.1016/j.cognition.2022.105250

[CR71] Schwarz, K. A., Pfister, R., Kluge, M., Weller, L., & Kunde, W. (2018). Do we see it or not? Sensory attenuation in the visual domain. *Journal of Experimental Psychology: General,**147*(3), 418–430. 10.1037/xge0000353 29154616 10.1037/xge0000353

[CR72] Schwarz, K. A., Weller, L., Klaffehn, A. L., & Pfister, R. (2019). The effects of action choice on temporal binding, agency ratings, and their correlation. *Consciousness and Cognition,**75*, 102807. 10.1016/j.concog.2019.102807 31494358 10.1016/j.concog.2019.102807

[CR73] Sebanz, N., & Lackner, U. (2007). Who’s calling the shots? Intentional content and feelings of control. *Consciousness and Cognition,**16*(4), 859–876. 10.1016/j.concog.2006.08.002 17045811 10.1016/j.concog.2006.08.002

[CR74] Seth, A. K. (2014). A predictive processing theory of sensorimotor contingencies: Explaining the puzzle of perceptual presence and its absence in synesthesia. *Cognitive Neuroscience,**5*(2), 97–118. 10.1080/17588928.2013.877880 24446823 10.1080/17588928.2013.877880 PMC4037840

[CR75] Sidarus, N., Vuorre, M., & Haggard, P. (2017). How action selection influences the sense of agency: An ERP study. *NeuroImage,**150*, 1–13. 10.1016/j.neuroimage.2017.02.015 28188916 10.1016/j.neuroimage.2017.02.015

[CR76] Singmann, H., Bolker, B., Westfall, J., Aust, F., & Ben-Shachar, M. S. (2023). *afex: Analysis of factorial experiments. R package version 1.3-0*. https://CRAN.R-project.org/package=afex

[CR77] Slamecka, N. J., & Graf, P. (1978). The generation effect: Delineation of a phenomenon. *Journal of Experimental Psychology: Human Learning and Memory,**4*(6), 592–604. 10.1037/0278-7393.4.6.592

[CR78] Synofzik, M., Vosgerau, G., & Newen, A. (2008). Beyond the comparator model: A multifactorial two-step account of agency. *Consciousness and Cognition,**17*(1), 219–239. 10.1016/j.concog.2007.03.010 17482480 10.1016/j.concog.2007.03.010

[CR79] Tsuji, N., & Imaizumi, S. (2022). Sense of agency may not improve recollection and familiarity in recognition memory. *Scientific Reports,**12*(1), 21711. 10.1038/s41598-022-26210-1 36522458 10.1038/s41598-022-26210-1 PMC9755117

[CR80] Tulving, E. (1985). Memory and consciousness. *Canadian Psychology/Psychologie Canadienne,**26*(1), 1–12. 10.1037/h0080017

[CR81] van den Bos, M., Cunningham, S. J., Conway, M. A., & Turk, D. J. (2010). Mine to remember: The impact of ownership on recollective experience. *Quarterly Journal of Experimental Psychology,**63*(6), 1065–1071. 10.1080/17470211003770938 10.1080/17470211003770938 20401814

[CR82] Verschoor, S. A., & Hommel, B. (2017). Self-by-doing: The role of action for self-acquisition. *Social Cognition,**35*(2), 127–145. 10.1521/soco.2017.35.2.127

[CR83] Villa, R., Tidoni, E., Porciello, G., & Aglioti, S. M. (2021). Freedom to act enhances the sense of agency, while movement and goal-related prediction errors reduce it. *Psychological Research,**85*(3), 987–1004. 10.1007/s00426-020-01319-y 10.1007/s00426-020-01319-y 32236696

[CR84] Võ, M. L. H., Conrad, M., Kuchinke, L., Urton, K., Hofmann, M. J., & Jacobs, A. M. (2009). The Berlin Affective Word List Reloaded (BAWL-R). *Behavior Research Methods,**41*(2), 534–538. 10.3758/BRM.41.2.534 10.3758/BRM.41.2.534 19363195

[CR85] Wegner, D. M. (2003). The mind’s best trick: How we experience conscious will. *Trends in Cognitive Sciences,**7*(2), 65–69. 10.1016/S1364-6613(03)00002-0 12584024 10.1016/s1364-6613(03)00002-0

[CR86] Wegner, D. M., & Wheatley, T. (1999). Apparent mental causation: Sources of the experience of will. *American Psychologist,**54*(7), 480–492. 10.1037/0003-066X.54.7.480 10424155 10.1037//0003-066x.54.7.480

[CR87] Wen, W., & Haggard, P. (2018). Control changes the way we look at the world. *Journal of Cognitive Neuroscience,**30*(4), 603–619. 10.1162/jocn_a_01226 29308984 10.1162/jocn_a_01226

[CR88] Wenke, D., Fleming, S. M., & Haggard, P. (2010). Subliminal priming of actions influences sense of control over effects of action. *Cognition,**115*(1), 26–38. 10.1016/j.cognition.2009.10.016 19945697 10.1016/j.cognition.2009.10.016

[CR89] Yebra, M., Galarza-Vallejo, A., Soto-Leon, V., Gonzalez-Rosa, J. J., de Berker, A. O., Bestmann, S., ... Strange, B. A. (2019). Action boosts episodic memory encoding in humans via engagement of a noradrenergic system. *Nature Communications,**10*(1), 3534. 10.1038/s41467-019-11358-8 10.1038/s41467-019-11358-8 PMC6684634 31388000

[CR90] Yonelinas, A. P. (2002). The nature of recollection and familiarity: A review of 30 years of research. *Journal of Memory and Language,**46*(3), 441–517. 10.1006/jmla.2002.2864

